# Intraoperative traction in posterior spinal fusion for adolescent idiopathic scoliosis: an updated systematic review and meta-analysis of correction, perioperative, and safety outcomes

**DOI:** 10.3389/fped.2026.1868371

**Published:** 2026-09-11

**Authors:** Qiang Tang, Rong Xu, Lijun Zhang, Hua Liu, Weihong Wang

**Affiliations:** 1 Department of Pediatric Surgery, Kunshan Women and Children's Health Care Hospital, Kunshan, China; 2 Department of Neurosurgery, Affiliated Kunshan Hospital of Jiangsu University, Kunshan, China

**Keywords:** adolescent idiopathic scoliosis, halo-femoral traction, intraoperative traction, meta-analysis, neuromonitoring, posterior spinal fusion

## Abstract

**Background:**

Intraoperative traction is used during posterior spinal fusion or posterior spinal instrumentation for adolescent idiopathic scoliosis (AIS), but the comparative evidence is still limited and clinically heterogeneous. Previous reviews have also varied in whether they combined preoperative traction, traction radiographs, severe-AIS technical cohorts, and neuromonitoring studies with direct intraoperative traction-vs.-nontraction comparisons.

**Methods:**

This systematic review and meta-analysis followed the PRISMA 2020 statement and was registered in PROSPERO (CRD42023432276). PubMed via the National Library of Medicine, Web of Science Core Collection via Clarivate, and Embase via Elsevier were searched from inception to February 22, 2026. The core quantitative synthesis was limited to comparative AIS studies that evaluated intraoperative traction vs. no intraoperative traction during posterior spinal fusion or posterior spinal instrumentation. Severe-AIS, traction-weight, resource-utilization, and neuromonitoring studies were summarized separately. Continuous outcomes were pooled as mean differences (MDs), and dichotomous outcomes as risk ratios (RRs). Random-effects models with Hartung-Knapp adjustment were used for the primary inference.

**Results:**

Eighteen studies were included in the systematic review, and five direct comparative studies contributed to the core meta-analysis. Intraoperative traction showed a favorable but imprecise association with correction rate (4 studies; 450 participants; MD, 5.54 percentage points; 95% CI, −0.18 to 11.27; *p* = 0.054; *I*^2^ = 56.3%). No clear difference was observed in number of fused levels, operative time, or blood loss. Transfusion results favored traction in direction but were based on only two studies and were very imprecise under Hartung-Knapp inference (RR, 0.48; 95% CI, 0.03–6.98). Overall complications and revision surgery showed no clear difference. Neuromonitoring events were reported more often in traction-related settings, especially with higher traction weights, larger or stiffer curves, and high-risk spinal cord morphology; most events recovered after traction reduction or release.

**Conclusions:**

Direct comparative evidence did not demonstrate a statistically significant improvement in coronal correction under Hartung-Knapp inference, and the available data were insufficient to establish a reduction in transfusion. The correction point estimate favored traction, but the finding is exploratory and the certainty of evidence is very low because of sparse comparative data, heterogeneity, imprecision, and residual confounding. Reversible intraoperative neuromonitoring alerts should be distinguished from permanent postoperative neurological deficits.

## 1 Introduction

Adolescent idiopathic scoliosis (AIS) is the most common spinal deformity requiring operative treatment during adolescence. For progressive curves that exceed accepted surgical thresholds, or for deformities that are clinically meaningful despite nonoperative care, posterior spinal fusion or posterior spinal instrumentation remains a standard corrective approach (1, 2). Surgical aims are common but challenging to achieve: to correct coronal and rotational deformity, to maintain proper sagittal alignment, to not fuse additional level, and to decrease perioperative and neurological risks (3). In such a context, intraoperative traction has been applied as a supplement to slowly decrease the deformity prior to final correction and to minimize the magnitude of force required in translation of rod, rod rotation, compression-distraction, or sequential instrumentation (4–6).

A practical difficulty is that intraoperative traction is not one single technique. Published AIS studies have described head halter traction with lower-extremity skin traction, intraoperative halo-femoral traction (IOHFT), intraoperative skull-femoral traction (IOSFT), intraoperative skull-skeletal traction (ISST), cranio-femoral traction (CFT), and posterior temporary traction (7). The methods vary in terms of their fix point, traction weight, time of application and release, use of skeletal pins, relation to neuromonitoring baseline, and the intended role during correction (8, 9). This similar terminology has also been applied in research on moderate AIS, severe rigid AIS, traction-weight protocols, neuromonitoring risk factors and technical correction strategies (10, 11). The pooled outcome could look statistically tidy but would not address any clear clinical question if all these reports were considered as the same intervention.

The usefulness of intraoperative traction in posterior AIS surgery remains a controversial issue (12). The potential benefits may be partial pre-instrumentation correction, simpler exposure, better rod seating, decreased mechanical forces on implants, less blood loss or blood transfusion needs, and in some severe cases, fewer extensive releases are required (13, 14). Conversely, traction is technical, needs equipment and team coordination, might cause pin- or traction-site complications, and may lead to motor evoked potential (MEP) changes or intraoperative neuromonitoring (IONM) changes (15, 16). The given safety outcomes should be separated carefully. Postoperative complication, traction related complication, reversible IONM warning, and permanent neurological deficit are not similar clinical events (17, 18). Combining them will either underestimate the physiological risk of traction or overestimate its clinical consequence.

Earlier traction literature has provided useful observations, but direct comparative evidence for intraoperative traction vs. nontraction during posterior AIS fusion remains sparse. Some studies reported better correction or lower resource use, while others found little additional benefit once baseline curve severity, flexibility, institutional protocols, and surgical strategy were considered (19). More recent neuromonitoring studies also suggest that the safety signal may not be uniform across all patients. Higher traction weight, larger or stiffer curves, and high-risk spinal cord morphology appear to be relevant contexts (20). For this reason, an updated review should separate the core efficacy question from related but different technical and safety questions.

This systematic review and meta-analysis addressed a focused PICOS question: in AIS patients undergoing posterior spinal fusion or posterior spinal instrumentation, how does intraoperative traction compare with no intraoperative traction for correction, perioperative, and safety outcomes? Direct traction-vs.-nontraction AIS studies were included in the core quantitative synthesis. Severe-AIS technical studies, traction-weight comparisons, sagittal balance or resource-utilization studies, and neuromonitoring risk-factor cohorts were summarized separately (21). The aim was not to present traction as a settled intervention, but to clarify what the current evidence can support, what should remain descriptive, and where important uncertainty remains.

## 2 Methods

### 2.1 Protocol and registration

This review was conducted according to the PRISMA 2020 statement (22). The protocol has been registered in PROSPERO beforehand with registration number CRD42023432276. The search update was performed as of February 22, 2026. Review question, eligibility criteria, evidence-layer structure, and outcome hierarchy were agreed upon prior to final synthesis. Since various more modern neuromonitoring and severe-AIS technical articles asked similar but not identical questions, the analysis plan divided the core comparative studies and supplementary evidence rather than squeezing all eligible reports into a single pooled analysis.

### 2.2 Search strategy and information sources

The search was conducted in PubMed through the National Library of Medicine, Web of Science Core Collection through Clarivate and Embase through Elsevier as of the inception of the databases until February 22, 2026. Searches were made with a combination of controlled vocabulary and free-text terms to search the following: adolescent idiopathic scoliosis, posterior spinal fusion or posterior spinal instrumentation, posterior spinal arthrodesis, and intraoperative traction. Reference lists of eligible studies and relevant reviews were also screened manually. The database search identified 1,082 records, including 308 from PubMed, 397 from Web of Science, and 377 from Embase. During full-text review, unrelated uses of AIS, such as acute ischemic stroke, were excluded.

### 2.3 Eligibility criteria and evidence-layer structure

Eligibility criteria followed the PICOS framework. The population of interest was AIS patients undergoing posterior spinal fusion, posterior spinal instrumentation, posterior spinal arthrodesis, or posterior-only correction. Mixed-etiology studies were eligible for the core meta-analysis only when AIS-specific data could be extracted separately. Neuromuscular, congenital, syndromic, adult degenerative, and secondary scoliosis populations were excluded from the core synthesis unless AIS data were separable.

The intervention was intraoperative traction applied during posterior correction, including IOHFT, IOSFT, ISST, CFT, head halter traction with lower-extremity skin traction, or another traction approach clearly used during surgery to assist posterior AIS correction. The comparator for the core meta-analysis was no intraoperative traction or conventional posterior fusion/instrumentation without intraoperative traction. Studies in which both arms received traction, or in which traction was bundled with different surgical strategies in both arms, were excluded from the core meta-analysis and retained only as supplementary evidence when they remained clinically informative.

Correction outcomes included correction rate, postoperative or final Cobb angle, and major curve correction. Perioperative outcomes included number of fused levels, operative time, estimated blood loss, and transfusion. Safety outcomes included revision surgery, overall complications, postoperative neurological deficit, MEP/IONM alerts, and traction-related complications. Sagittal balance, spinopelvic parameters, SRS-22, pulmonary function, height gain, resource usage, and neuromonitoring recovery were considered to be supplementary outcomes. The core meta-analysis included randomized trials, prospective cohorts, retrospective cohorts, matched cohorts, and comparative observational studies if they compared intraoperative traction with no traction directly. Narrative synthesis contained only single-arm cohorts, case series, traction-weight studies, neuromonitoring-focused observational studies, and severe-AIS technical cohorts.

### 2.4 Study selection and data extraction

Two reviewers independently screened titles and abstracts, assessed full texts, and extracted data using a standardized form. Disagreements were resolved by discussion or, when needed, by consultation with a third reviewer. Extracted variables included author, publication year, country or region, study design, study period, population, curve severity, sample size, age, sex distribution, baseline Cobb angle, flexibility, follow-up, traction type, comparator, correction outcomes, perioperative outcomes, safety outcomes, neuromonitoring findings, and risk-of-bias information. When reports were derived from overlapping cohorts, the most clinically appropriate or least overlapping data source was used for quantitative synthesis, while related reports were described narratively. The final evidence classification and baseline profile are shown in Table 1.

**Table 1:** Characteristics and baseline profile of included studies.

Study ID	Study	Region	Design	Population and curve severity	Sample size	Age	Sex	Baseline curve and flexibility	Follow-up	Traction/comparator	Evidence role
S001	Mac-Thiong et al. (7)	Canada	Retrospective comparative cohort	AIS undergoing posterior spinal instrumentation/fusion; moderate-to-severe mixed curves	40 traction/100 control	15.2 ± 2.0 vs. 15.3 ± 2.0 years	100.0% vs. 93.0% female	Primary Cobb 55.2 ± 9.1° vs. 61.5 ± 11.6°; primary-curve flexibility 50.8 ± 17.2% vs. 44.7 ± 19.3%	1 month postoperative; lateral radiographs at 1 year	Head halter plus lower-extremity skin traction vs. no traction	Core meta-analysis; sensitivity analysis because traction was non-skeletal
S002	Da Cunha et al. (8)	Canada	Retrospective cohort	AIS undergoing posterior spinal arthrodesis	45 IOSFT/28 control	14.6 (10–18) vs. 14.7 (12–18) years	91.1% vs. 92.9% female	Major Cobb 62.0° (45–95) vs. 63.6° (42–81); flexibility NR	Minimum 6 months for correction outcomes	IOSFT vs. no IOSFT	Core meta-analysis; continuous outcomes limited by non-SD reporting
S003	Hu et al. (9)	China	Randomized controlled trial	Severe rigid AIS, 70°-100°, flexibility <35%	15 IOHFT/14 control	14.80 ± 1.97 vs. 14.93 ± 1.77 years	80.0% vs. 85.7% female	83.20 ± 7.42° vs. 80.29 ± 7.54°; flexibility 20.63 ± 6.80% vs. 22.84 ± 6.58%	27.93 ± 2.87 vs. 27.36 ± 2.50 months	IOHFT vs. no traction	Core meta-analysis; randomized trial
S004	Rushton et al. (13)	North America	Matched cohort	Major thoracic AIS, Lenke 1–4	104 IOT/104 non-IOT	15.2 ± 1.8 vs. 15.2 ± 1.7 years	91.0% vs. 77.0% female	60.6 ± 11.3° vs. 60.8 ± 11.2°; flexibility 30.0 ± 15.9% vs. 31.1 ± 14.7%	2 years	IOT vs. matched non-IOT	Core meta-analysis
S005	Carl et al. (14)	United States	Retrospective comparative study	AIS posterior-only fixation, curves 50°-90°	37 CFT/36 control	14.6 ± 1.6 vs. 15.4 ± 2.3 years	NR	64.0 ± 9.9° vs. 58.0 ± 8.9°; flexibility 61.0 ± 14.5% vs. 60.3 ± 16.8%	Initial postoperative assessment before discharge	CFT vs. no CFT	Core meta-analysis; operative-time data available for 37 traction/31 control
S006	Peiro-Garcia et al. (25)	Canada	Retrospective supplementary cohort	AIS undergoing single-stage posterior spinal instrumentation; sagittal-balance focus	85 IOSFT/28 control	Not reported	Not reported	NR	2 years	IOSFT context vs. no traction context	Supplementary sagittal/spinopelvic evidence
S007	Bourget-Murray et al. (26)	Canada	Retrospective resource-utilization cohort	AIS posterior spinal instrumentation/fusion; overlapping Alberta program	Cohort A 28; Cohort B 45; Cohort C 52	Not reported	Not reported	Preoperative Cobb differed across cohorts; exact values not used for core pooling	Minimum 6 months	No IOSFT/no NSD vs. IOSFT vs. IOSFT plus NSD	Supplementary resource-utilization evidence; not pooled because of overlap with S002
S008	Lewis et al. (15)	Canada	Neuromonitoring-focused observational study	AIS deformity correction with IOSFT; severe curves enriched	37 IOSFT procedures/35 no-traction comparison	14.8 years mean in traction series	27F/9M among 36 patients	MEP-change group 86°; no-change group 70°; control group 59°; flexibility index 0.14 vs. 0.27 vs. 0.25	Perioperative	IOSFT procedures vs. no-traction comparison	Supplementary MEP/SSEP safety evidence
S009	Kato et al. (16)	Canada	Retrospective traction-weight comparison	AIS posterior spinal fusion	38 high-weight ISST/42 low-weight ISST/36 no ISST	15 vs. 14 vs. 15 years	74.0% vs. 69.0% vs. 92.0% female	77° vs. 69° vs. 57°; flexibility 23% vs. 29% vs. 23%	Postoperative radiographs	High-weight ISST vs. low-weight ISST vs. no ISST	Supplementary traction-weight safety/correction evidence
S010	Berends et al. (17)	Netherlands	Risk-factor cohort	AIS and neuromuscular scoliosis undergoing deformity surgery	81 total; AIS subgroup 47	15.6 ± 2.4 years	53 females overall	Median 60° overall; AIS 59°; neuromuscular scoliosis 67°; flexibility 34.8 ± 14.5% overall	Perioperative	IOHFT-related MEP event assessment	Supplementary neuromonitoring safety evidence
S011	Hadden et al. (18)	United States	Risk-factor cohort	AIS major thoracic curves ≥70° with preoperative MRI	15 traction/87 no traction	13.7 ± 2.0 years overall	75/102 female	85.7 ± 13.1° overall; flexibility not reported	Perioperative	Intraoperative traction vs. no traction; spinal cord morphology assessment	Supplementary neuromonitoring safety evidence
S012	Erdem et al. (4)	Turkey	Retrospective single-arm clinical study	Severe AIS, 70°-90°, flexibility <35%	24 single-arm	17.4 years	18F/6M	80.1° (range 70–90°); flexibility 21.1% (range 11.4%–33.1%)	33.5 months	Intraoperative HFT; no control group	Supplementary severe-AIS technical evidence
S013	Jhaveri et al. (5)	Canada	Retrospective consecutive case series	Pediatric scoliosis; mixed AIS and neuromuscular scoliosis	22 total; AIS 14/neuromuscular scoliosis 8	Not reported	Not reported	Mean curve 88.2° (range 55–132°); large/inflexible curves	Intraoperative/postoperative	Intraoperative skull-skeletal traction; no control group	Supplementary mechanistic/AVR evidence
S014	Zhang et al. (6)	China	Retrospective comparative technical cohort	AIS curves >100°	17 posterior-only HFT/12 combined anterior-posterior	15.7 vs. 15.2 years	12F/5M vs. 9F/3M	107.6 ± 7.9° vs. 106.3 ± 8.5°; flexibility 31% vs. 32%	Mean 36 months	Posterior-only surgery plus strong HFT vs. combined anterior-posterior strategy with HFT	Supplementary severe-AIS strategy evidence; not traction vs. nontraction
S015	Zhang et al. (10)	China	Prospective comparative/quasi-randomized by hospitalization month	Severe rigid AIS, >80°, flexibility <35%	33 posterior-only/31 combined anterior-posterior	15.9 vs. 16.5 years	25F/8M vs. 24F/7M	85.4° vs. 83.6°; flexibility 31.8% vs. 33.2%	37.5 months	Posterior-only HFT strategy vs. combined anterior-posterior HFT strategy	Supplementary severe-AIS strategy evidence; not a standard RCT
S016	Zhang et al. (11)	China	Retrospective single-arm technical cohort	Extremely severe rigid AIS >130°	11 single-arm	13.36 ± 1.57 years	7F/4M	139.01 ± 5.83°; flexibility 17.21 ± 3.33%	32.18 ± 8.17 months	Preoperative heavy HFT for 3–6 weeks plus intraoperative maintained HFT; no control group	Supplementary extremely severe-AIS technical evidence
S017	Kulkarni et al., (12)	India	Retrospective comparative technical study	High-magnitude AIS curves >65°, flexibility index <0.5	10 IOT/14 IOT plus Ponte osteotomy	19.4 vs. 16.8 years	Not reported	89.35 ± 6.05° vs. 92.32 ± 9.28°; flexibility index 0.31 ± 0.016 vs. 0.36 ± 0.03	2 years	IOT alone vs. IOT plus Ponte osteotomy	Supplementary technique-comparison evidence; both arms received traction
S018	Acar et al. (19)	Turkey	Retrospective single-arm technical study	AIS treated with posterior instrumentation/fusion using temporary traction	18 single-arm	16.05 years	14F/4M	50.83° (range 30–72°); flexibility not reported	35.8 months	Posterior temporary traction with Ilizarov wire; no control group	Supplementary posterior temporary traction evidence

The table summarizes study design, evidence layer, sample size, age, sex distribution, baseline curve severity, flexibility, follow-up, and traction/comparator definitions. AIS, adolescent idiopathic scoliosis; AVR, apical vertebral rotation; CFT, cranio-femoral traction; HFT, halo-femoral traction; IOHFT, intraoperative halo-femoral traction; IOSFT, intraoperative skull-femoral traction; IOT, intraoperative traction; ISST, intraoperative skull-skeletal traction; MEP, motor evoked potential; MRI, magnetic resonance imaging; NSD, navigated sequential drilling; SSEP, somatosensory evoked potential. Values are presented as traction/comparator unless otherwise specified. NR, not reported.

### 2.5 Risk-of-bias assessment and certainty of evidence

Risk of bias was assessed according to study design. The randomized trial was assessed with the revised Cochrane risk-of-bias tool for randomized trials (RoB 2) (23). The four non-randomized comparative studies were assessed with ROBINS-I across bias due to confounding, selection of participants, classification of interventions, deviations from intended interventions, missing data, measurement of outcomes, and selection of the reported result (24). Because treatment assignment in observational studies could depend on baseline curve magnitude and flexibility, center or surgeon preference, treatment era, implant density, osteotomy or release strategy, or other aspects of case complexity, confounding by indication was treated as a key concern. Single-arm technical cohorts and case series in the supplementary synthesis were evaluated with JBI case-series domains. The certainty of evidence for key outcomes was assessed using the GRADE framework, considering risk of bias, inconsistency, indirectness, imprecision, publication bias, and other relevant factors.

### 2.6 Statistical analysis

Mean differences (MDs) were pooled as continuous outcomes and risk ratios (RRs) as dichotomous outcomes, both with 95% confidence intervals. The main model was random-effects with the adjustment of Hartung-Knapp. It was selected since it was anticipated that there would be clinical heterogeneity among the different methods of traction, severity of curves, design of the studies and their outcomes and also that there were only a few studies per any given outcome. Sensitivity analyses were done with fixed-effect models and conventional random-effects models not adjusted to Hartung-Knapp. Tests to assess heterogeneity included Cochran's *Q* test, tau-squared and *I*^2^ statistics.

Leave-one-out sensitivity analyses were conducted for correction rate, number of fused levels, operative time, and blood loss. S001/Mac-Thiong et al. was retained in the primary comparative evidence set because the prespecified intervention definition included intraoperative traction used during posterior AIS correction and did not require skeletal pin fixation; the study directly compared intraoperative traction with no traction. Because S001 used head halter plus lower-extremity skin traction, which differs mechanically from skeletal traction, an additional exclusion sensitivity analysis was performed to test intervention-definition heterogeneity. Subgroup analyses by traction type and curve severity were performed when sufficient data were available. Primary continuous-outcome analyses included studies with directly extractable means and standard deviations; studies without directly extractable standard deviations were described or evaluated only in supplementary sensitivity analyses. Funnel plots and Egger regression were treated as exploratory because fewer than 10 studies were available per pooled outcome. Analyses were performed in R version 4.5.2 using standard meta-analysis packages.

## 3 Results

### 3.1 Study selection

**Figure 1:**
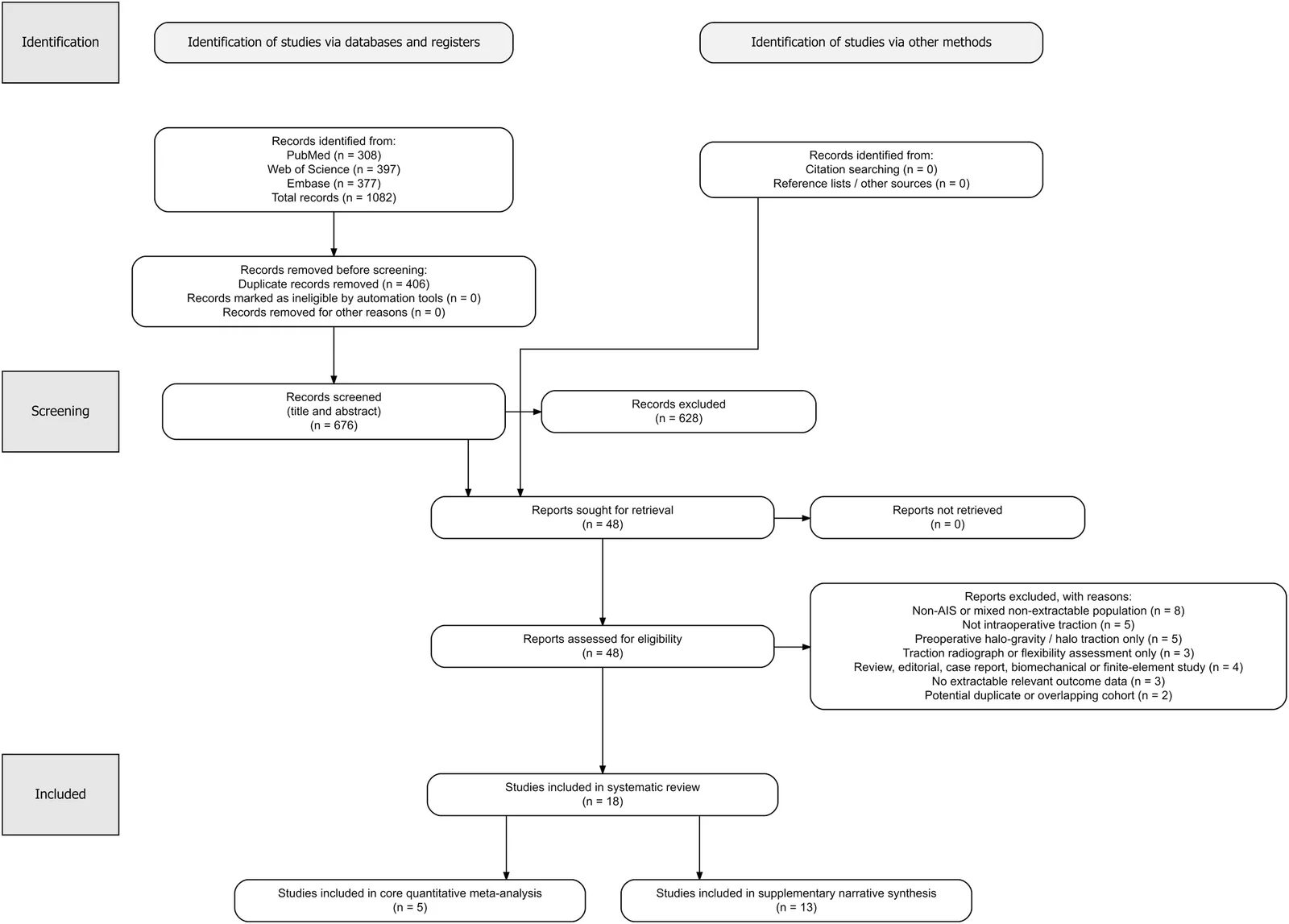
PRISMA flow diagram. The flow diagram shows database identification, duplicate removal, title/abstract screening, full-text assessment, and final inclusion. A total of 1,082 records were identified, 676 records were screened after duplicate removal, 48 reports underwent full-text assessment, 18 studies were included in the systematic review, five were included in the core quantitative meta-analysis, and 13 were included in supplementary narrative synthesis.

Figure 1 depicts the study-selection process. The search of the database yielded 1,082 records. After elimination of duplicates, 676 records were filtered by title and abstract. Full-text evaluation was performed on forty-eight reports and eighteen studies were selected to be included into the systematic review. Five direct comparative studies were integrated into the main quantitative meta-analysis whereas 13 studies were kept as supplementary narrative evidence. The supplementary evidence comprised sagittal-balance and resource utilization reports, studies focusing on neuromonitoring, weight-traction studies, and severe or extremely severe AIS technical cohorts.

### 3.2 Study characteristics and evidence layers

The five core comparative studies considered intraoperative traction during posterior AIS surgery compared to no traction. The methods of applying traction were head halter with lower-extremity skin traction, IOSFT, IOHFT, IOT, and CFT. The populations were not comparable: curve severity and flexibility were moderate-to-severe mixed AIS groups, severe rigid curves of 70–100, and AIS curves of 50–90 degrees. Follow up also differed and was done early after operation or at 2 years. Such differences justified the application of conservative random-effects inference instead of fixed-effect assumption.

The supplementary evidence was kept separate from the core synthesis. Peiro-Garcia et al. and Bourget-Murray et al. reported sagittal-balance or resource-utilization outcomes related to the Alberta IOSFT experience, but their outcomes, summary formats, and cohort overlap made routine pooling with Da Cunha et al. inappropriate. Lewis et al., Kato et al., Berends et al., and Hadden et al. provided neuromonitoring or traction-weight safety evidence. Erdem et al., Jhaveri et al., Zhang et al., Zhang/Deng et al., Kulkarni et al., and Acar et al. addressed severe-AIS technical or mechanistic questions, often without a no-traction comparator or with both arms receiving traction. S015 was classified as a prospective comparative/quasi-randomized study by hospitalization month rather than a standard randomized trial. Koptan and ElMiligui (27) and Sponseller et al. (28) were not included in the core meta-analysis because they addressed limited or preoperative halo-gravity traction rather than direct intraoperative traction vs. nontraction during posterior AIS fusion.

### 3.3 Risk of bias and certainty of evidence

Risk-of-bias findings are summarized in Supplementary Figure S4 and Supplementary Tables S1, S2. The randomized trial was judged as having some concerns under RoB 2, principally because allocation-concealment and selective-reporting details were limited, although outcome data were complete and radiographic assessment was independent. All four non-randomized comparative studies were judged at serious overall risk of bias under ROBINS-I, driven mainly by residual confounding. In S001, traction was used according to surgeon preference and the groups differed in baseline Cobb angle, flexibility, fusion length, screw use, and instrumentation. S002 used a historical before-after implementation pattern, with all traction cases treated in 2010–2012 and most controls in 2008–2009. S004 used matching, but center, sex, thoracic apical translation, traction technique, and metal-density differences remained. In S005, the traction group had larger baseline curves and treatment spanned a long period during which surgeon experience evolved. The GRADE assessment therefore rated the certainty of the principal comparative outcomes as very low, reflecting serious risk of bias together with inconsistency and/or substantial imprecision (Supplementary Table S3).

### 3.4 Main quantitative synthesis

**Figure 2:**
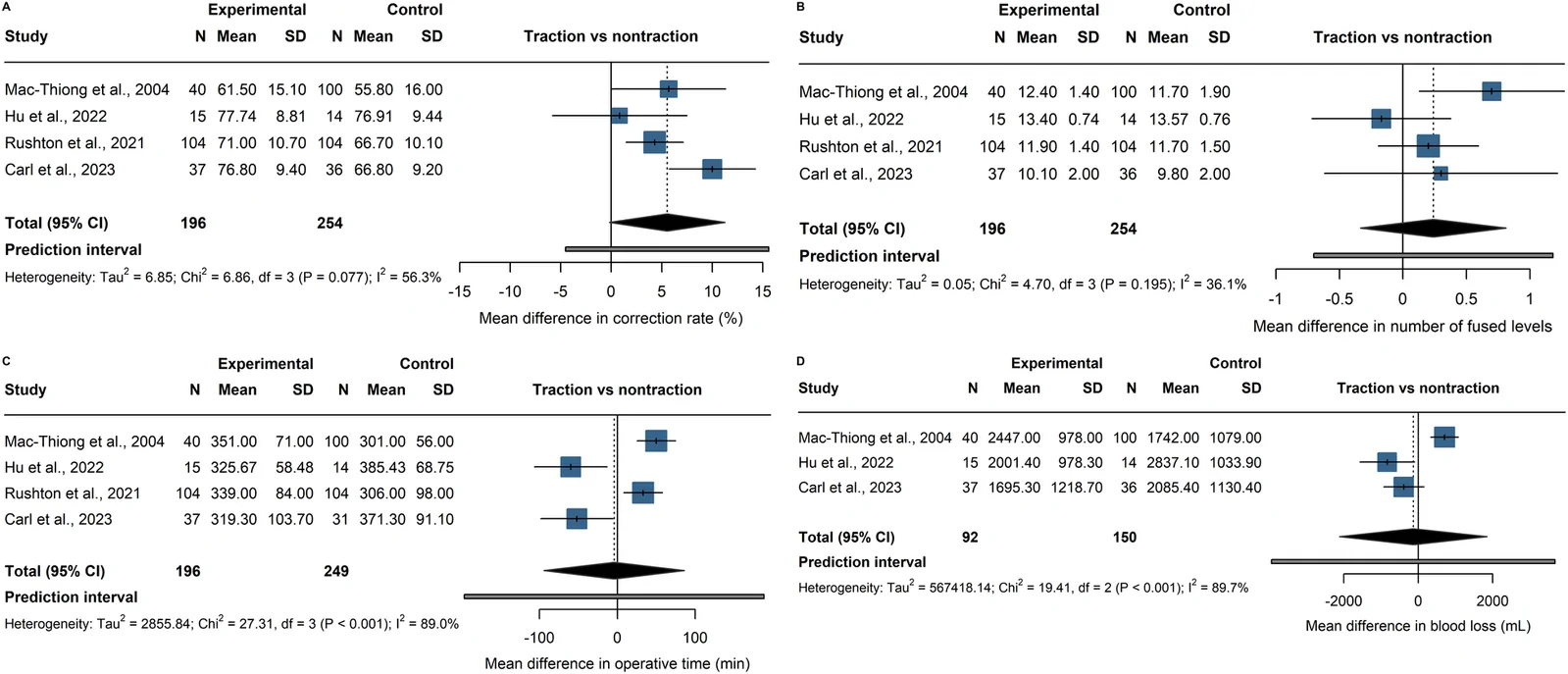
Main continuous outcome meta-analyses. Forest plots show mean differences comparing intraoperative traction with nontraction for **(A)** correction rate, **(B)** number of fused levels, **(C)** operative time, and **(D)** blood loss. The primary pooled model was a random-effects model with Hartung-Knapp adjustment.

The main pooled estimates are summarized in Table 2. For correction rate, four studies with 450 participants reported extractable mean and standard deviation data. The point estimate favored intraoperative traction, but direct comparative evidence did not demonstrate a statistically significant improvement under Hartung-Knapp inference because the confidence interval crossed the null (MD, 5.54 percentage points; 95% CI, −0.18 to 11.27; *p* = 0.054; *I*^2^ = 56.3%) (Figure 2A). Accordingly, this result is exploratory rather than evidence of established superiority.

**Table 2:** Main meta-analysis results under the hartung-knapp random-effects model.

Outcome	Studies	Participants	Effect measure	Pooled estimate	HK 95% CI	*p* value	*I* ^2^	GRADE certainty	Interpretation
Correction rate	4	450 (196/254)	MD	5.54 percentage points	−0.18 to 11.27	0.054	56.3%	Very low	No statistically significant benefit under HK inference; point estimate favors traction
Number of fused levels	4	450 (196/254)	MD	0.24 levels	−0.33 to 0.81	0.276	36.1%	Very low	No clear difference
Operative time	4	445 (196/249)	MD	−4.13 min	−94.28 to 86.01	0.893	89.0%	Very low	No clear difference; high heterogeneity
Blood loss	3	242 (92/150)	MD	−133.68 mL	−2,116.54 to 1,849.19	0.799	89.7%	Very low	No clear difference; high heterogeneity
Transfusion	2	102 (60/42)	RR	0.48	0.03 to 6.98	0.178	0.0%	Very low	Insufficient evidence to establish a transfusion reduction; HK CI extremely imprecise
Revision surgery	2	102 (60/42)	RR	0.52	0.01 to 25.54	0.277	0.0%	Very low	Sparse events; no clear difference; estimate very imprecise
Overall complications	3	242 (100/142)	RR	0.79	0.06 to 10.55	0.735	30.3%	Very low	Exploratory broad safety endpoint; no clear difference and very imprecise

Continuous outcomes are summarized as mean differences and dichotomous outcomes as risk ratios. CI, confidence interval; FE, fixed effect; GRADE, grading of recommendations assessment, development and evaluation; HK, Hartung-Knapp; *I*^2^, inconsistency statistic; MD, mean difference; RE, random effects; RR, risk ratio. Participant counts are shown as total (traction/control).

No clear difference was observed in number of fused levels (4 studies; 450 participants; MD, 0.24; 95% CI, −0.33 to 0.81; *p* = 0.276; *I*^2^ = 36.1%) (Figure 2B). Operative time also showed no clear difference under the primary model (4 studies; 445 participants; MD, −4.13 min; 95% CI, −94.28 to 86.01; *p* = 0.893; *I*^2^ = 89.0%) (Figure 2C). Blood loss showed no clear pooled reduction and substantial heterogeneity (3 studies; 242 participants; MD, −133.68 mL; 95% CI, −2,116.54 to 1,849.19; *p* = 0.799; *I*^2^ = 89.7%) (Figure 2D). These highly heterogeneous perioperative endpoints are likely to reflect differences beyond traction itself, including curve severity and rigidity, Ponte or other release procedures, implant density, cell-saver practice, tranexamic acid use, anesthetic and blood-pressure management, transfusion thresholds, surgeon experience, and institutional workflow.

**Figure 3:**
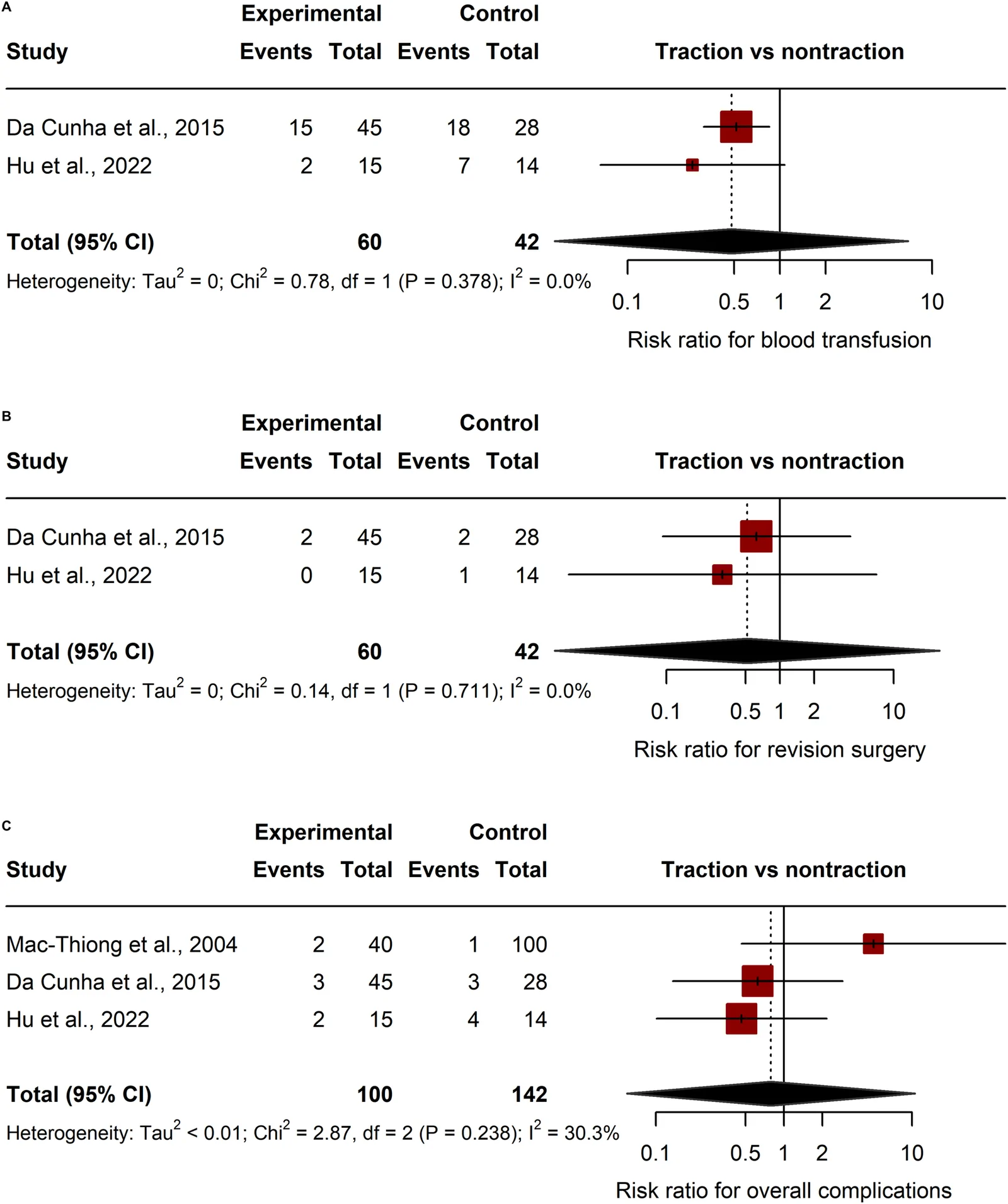
Dichotomous outcome meta-analyses. Forest plots show risk ratios comparing intraoperative traction with nontraction for **(A)** transfusion, **(B)** revision surgery, and **(C)** overall complications. Overall complications were treated as an exploratory broad safety endpoint because definitions differed across studies.

For dichotomous outcomes, two transfusion studies favored traction in direction, but the Hartung-Knapp-adjusted estimate was extremely imprecise because only two studies were available (RR, 0.48; 95% CI, 0.03–6.98; *p* = 0.178; *I*^2^ = 0.0%) (Figure 3A). Thus, the direct evidence does not establish a reduction in transfusion requirements. Revision surgery was rare, with no clear difference between groups (RR, 0.52; 95% CI, 0.01–25.54; *p* = 0.277; *I*^2^ = 0.0%) (Figure 3B). Overall complications were treated as an exploratory broad safety endpoint because definitions varied across studies; no clear increase was observed (3 studies; RR, 0.79; 95% CI, 0.06–10.55; *p* = 0.735; *I*^2^ = 30.3%) (Figure 3C). These estimates should be interpreted as sparse and imprecise rather than as evidence of equivalence or safety.

### 3.5 Subgroup and sensitivity analyses

Subgroup and sensitivity analyses are summarized in Table 3. Model comparisons showed that fixed-effect and conventional random-effects models often produced narrower confidence intervals than the Hartung-Knapp model, especially for correction rate and transfusion. For this reason, primary interpretation was based on Hartung-Knapp estimates. S001/Mac-Thiong et al. was retained in the primary analysis because it met the prespecified functional definition of intraoperative traction and provided a direct traction-vs.-no-traction comparison; however, its head-halter/lower-extremity skin traction differs from skeletal traction in force transmission. Excluding S001 therefore served as an intervention-definition sensitivity analysis. The correction-rate estimate remained directionally similar after exclusion (MD, 5.40; 95% CI, −5.57 to 16.37; *p* = 0.168), but precision decreased (Supplementary Figure S1). Operative time and blood loss also remained unstable, indicating that uncertainty in perioperative outcomes was not driven by S001 alone.

**Table 3:** Subgroup and sensitivity analyses.

Analysis	Studies	Estimate or result	*p* value	Interpretation
Correction rate: FE/RE-classic/RE-HK	4	FE: 5.49 (3.42–7.56); RE-classic: 5.54 (2.07–9.01); RE-HK: 5.54 (−0.18 to 11.27)	HK *p* = 0.054	Primary inference based on RE-HK
Fused levels: FE/RE-classic/RE-HK	4	FE: 0.23 (−0.04 to 0.50); RE-classic: 0.24 (−0.12 to 0.60); RE-HK: 0.24 (−0.33 to 0.81)	HK *p* = 0.276	No clear difference
Operative time: FE/RE-classic/RE-HK	4	FE: 20.11 (4.69–35.53); RE-classic: −4.13 (−59.60 to 51.33); RE-HK: −4.13 (−94.28 to 86.01)	HK *p* = 0.893	High heterogeneity; inference based on RE-HK
Blood loss: FE/RE-classic/RE-HK	3	FE: 179.62 (−101.86 to 461.11); RE-classic: −133.68 (−1,044.87 to 777.51); RE-HK: −133.68 (−2,116.54 to 1,849.19)	HK *p* = 0.799	High heterogeneity
Transfusion: FE/RE-classic/RE-HK	2	FE: 0.46 (0.28–0.74); RE-classic: 0.48 (0.30–0.77); RE-HK: 0.48 (0.03–6.98)	HK *p* = 0.178	Direction favors traction; HK CI imprecise
Correction rate excluding S001	3	MD 5.40 (−5.57 to 16.37)	0.168	S001 met the prespecified intraoperative-traction definition and was retained in the primary analysis; exclusion tested heterogeneity from its non-skeletal head-halter/skin-traction method
Operative time excluding S001	3	MD −23.38 (−153.94 to 107.18)	0.522	No clear difference
Blood loss excluding S001	2	MD −546.28 (−3,247.72 to 2,155.15)	0.236	No clear difference; very imprecise
Correction rate by traction type	4	Subgroup-difference chi-square=2.02, df = 2	0.365	Exploratory only
Correction rate by curve severity	4	Subgroup-difference chi-square=6.86, df = 3	0.077	Exploratory; mostly single-study categories
Leave-one-out correction rate	4	Omitted-study estimates ranged from 4.11 to 6.49	See Supplementary Figure S2	Direction remained favorable
Leave-one-out perioperative outcomes	3–4	Fused levels: 0.10–0.37; operative time: −23.38 to 13.24; blood loss: −546.28 to 175.75	See Supplementary Figure S2	Perioperative estimates were less stable

Model comparisons, S001-exclusion analyses, exploratory subgroups, and leave-one-out summaries are presented. CI, confidence interval; FE, fixed effect; HK, Hartung-Knapp; MD, mean difference; RE, random effects. Subgroup and leave-one-out analyses were exploratory because the number of studies was small. S001 was retained in the primary analysis because it met the prespecified functional intervention definition; its exclusion was an intervention-definition sensitivity analysis.

**Figure 4:**
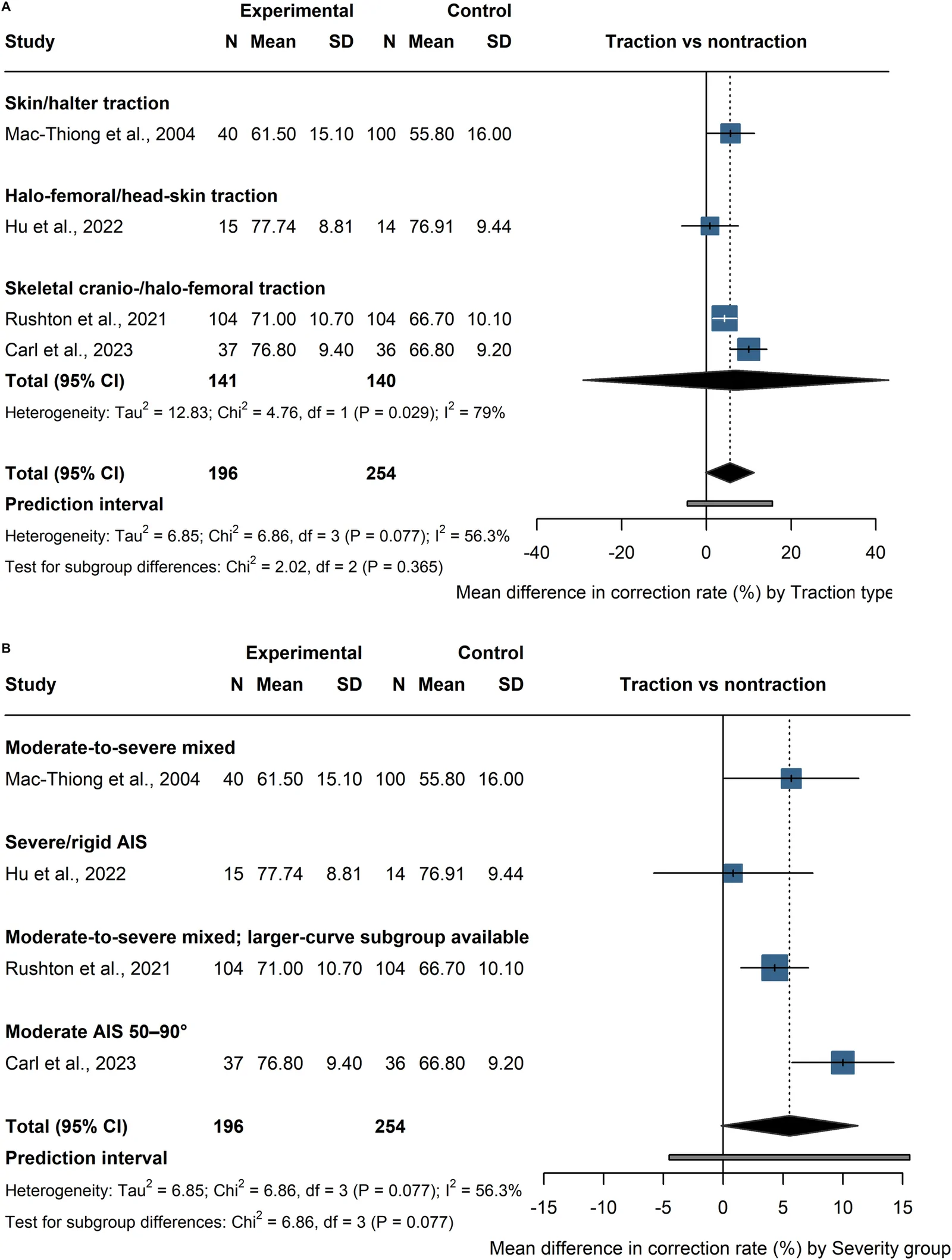
Exploratory correction-rate subgroup analyses. Subgroup forest plots show correction-rate mean differences by **(A)** traction type and **(B)** curve severity. These analyses were exploratory and should not be interpreted as definitive effect modification.

Exploratory subgroup analyses did not show a clear subgroup difference by traction type (*p* = 0.365) and suggested only borderline heterogeneity by curve severity (*p* = 0.077) (Figure 4). These findings should not be interpreted as evidence of definite effect modification because most subgroup categories contained only one study. Leave-one-out analyses showed that correction-rate estimates remained directionally favorable, whereas operative time and blood loss were more sensitive to individual studies (Supplementary Figure S2). Exploratory funnel plots are shown in Supplementary Figure S3. Because fewer than 10 studies were available for each pooled outcome, these analyses were underpowered and were not used to rule in or rule out publication bias.

### 3.6 Neuromonitoring and safety evidence

**Figure 5:**
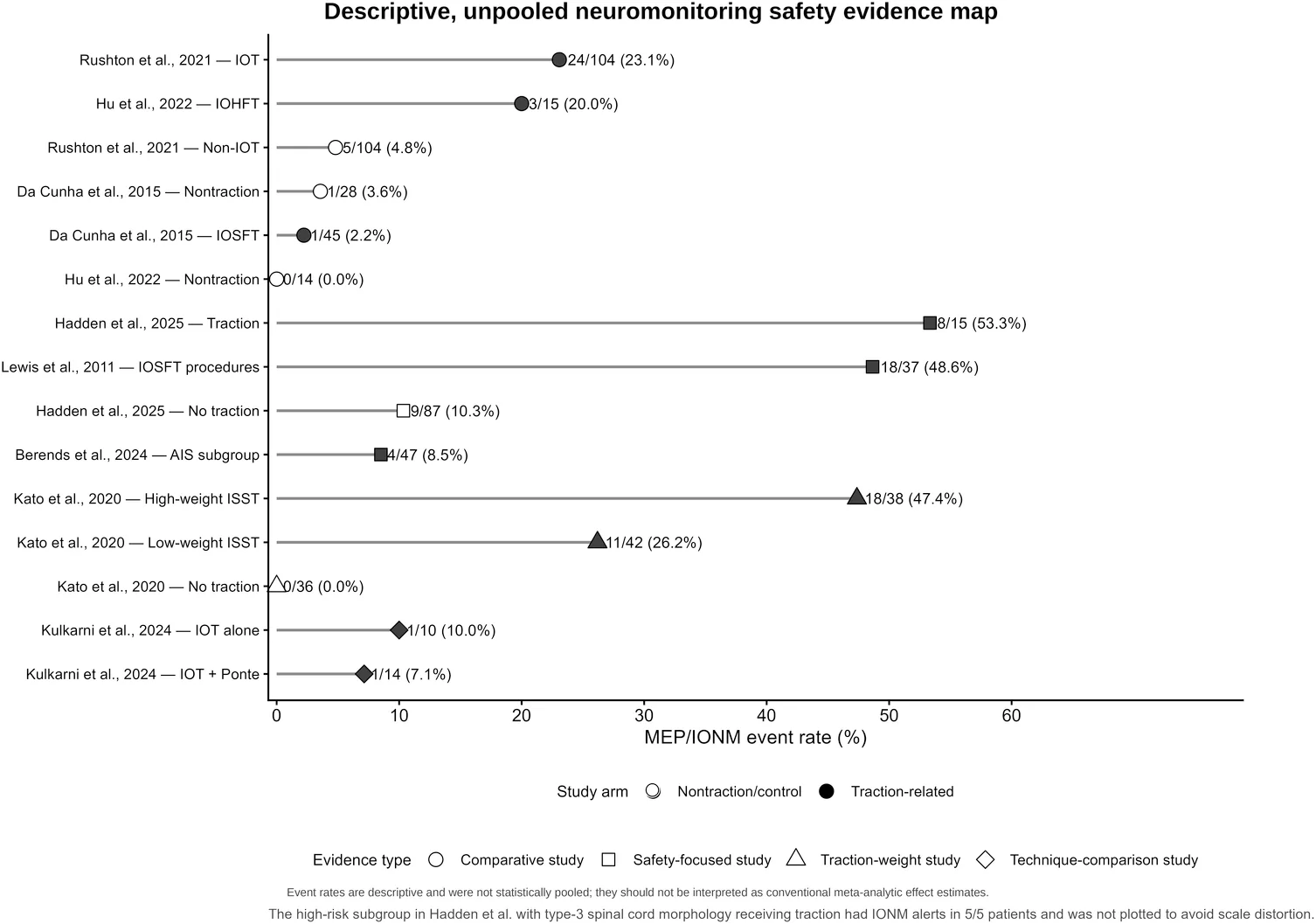
Descriptive, unpooled neuromonitoring safety evidence map. MEP/IONM event rates are shown across comparative and supplementary safety studies to illustrate the distribution of physiological warning events across different traction contexts. Because event definitions, denominators, traction protocols, and clinical risk profiles differed, these rates were not statistically pooled and should not be interpreted as conventional meta-analytic effect estimates. Permanent postoperative neurological deficits are conceptually distinct from reversible intraoperative alerts.

Neuromonitoring and safety evidence is summarized in Table 4 and Figure 5 as descriptive, unpooled evidence. The comparative studies themselves illustrate why physiological alerts and postoperative neurological deficits should be separated. In S002, one patient in each group had a temporary MEP decrease during main correction; releasing traction alone did not restore the signal in the traction case, whereas release of the correction and rod removal restored MEPs in both patients, and both had normal postoperative neurological examinations. In the randomized S003 study, MEP changes occurred in 3 of 15 traction patients and in 0 of 14 controls; all three signals recovered completely after traction release and no postoperative neurological deficits occurred. In S004, IONM alerts occurred in 24 of 104 IOT patients vs. 5 of 104 non-IOT patients; traction was a sole or combined precipitant in 11 of the 24 IOT alerts, and all 11 returned to baseline after traction reduction/removal with blood-pressure optimization. Supplementary safety studies similarly reported higher alert rates in traction-related or high-weight contexts. Hadden et al. reported alerts in 8 of 15 traction patients (53.3%) vs. 9 of 87 no-traction patients, and all five traction patients with type-3 spinal cord morphology had alerts. Across these reports, most traction-responsive alerts were reversible after corrective action, whereas permanent postoperative neurological deficits were uncommon. The small sample sizes and selected monitored settings, however, do not establish neurological safety in unmonitored or less standardized practice.

**Table 4:** Neuromonitoring and safety evidence summary.

Study/evidence group	Outcome/event	Events or rate	Recovery after traction reduction/release	Postoperative neurological deficit	Interpretation
S001 Mac-Thiong	Neurological complication	0/40 traction; 1/100 control	Not applicable	One control event	No direct traction-related neurological complication reported
S002 Da Cunha	MEP decrease	1/45 IOSFT; 1/28 control	Traction release alone did not restore the signal in the traction case; MEPs recovered in both patients after release of correction/rod removal	No postoperative neurological deficit in either patient	Temporary MEP changes during main correction; not a permanent neurological deficit signal
S003 Hu	MEP change	3/15 IOHFT; 0/14 control	All 3 MEP changes recovered completely after traction release	No postoperative neurological deficit	Small RCT; reversible physiological alerts should be separated from permanent deficit
S004 Rushton	IONM alert	24/104 IOT; 5/104 non-IOT	All 11 alerts in which traction was a sole or combined precipitant returned to baseline after traction reduction/removal plus blood-pressure optimization	No postoperative neurological deficits in either group	IONM alerts were more frequent with IOT (23.1% vs. 4.8%), but traction-attributed alerts were generally reversible
S008 Lewis	MEP changes	18/37 IOSFT procedures	Generally improved after reducing or removing traction	No postoperative deficit	Safety-focused IOSFT cohort
S009 Kato	MEP changes	18/38 high-weight ISST; 11/42 low-weight ISST; 0/36 no ISST	Recovered after decreasing traction weight	No neurological deficit	Higher traction weight was associated with more MEP changes
S010 Berends	IOHFT-related MEP events	11/81 overall; AIS subgroup 4/47	All recovered after removing IOHFT	No postoperative neurological impairment caused by IOHFT	Events associated with shorter stature and larger/stiffer curves
S011 Hadden	IONM alerts	8/15 traction; 9/87 no traction; type-3 cord + traction 5/5	Most alerts were reversible after corrective action	Permanent deficit was uncommon; small monitored cohorts do not establish safety	Traction-arm alert rate 53.3% (8/15); type-3 cord + traction 5/5; descriptive high-risk signal
S017 Kulkarni	SSEP events	1/10 IOT; 1/14 IOT + Ponte	Recovered	No postoperative deficit	Both arms received traction; not a core comparison

Event rates are summarized descriptively because definitions, denominators, and clinical contexts differed across studies. AIS, adolescent idiopathic scoliosis; IOHFT, intraoperative halo-femoral traction; IONM, intraoperative neuromonitoring; IOSFT, intraoperative skull-femoral traction; IOT, intraoperative traction; ISST, intraoperative skull-skeletal traction; MEP, motor evoked potential; RCT, randomized controlled trial; SSEP, somatosensory evoked potential. This table is descriptive and was not used for conventional meta-analysis. The event rates are descriptive and unpooled; reversible IONM/MEP alerts are not equivalent to permanent postoperative neurological deficits.

## 4 Discussion

This review provides a deliberately conservative synthesis of intraoperative traction during posterior AIS surgery. Under the primary Hartung-Knapp model, direct comparative evidence did not demonstrate a statistically significant improvement in coronal correction (MD, 5.54 percentage points; 95% CI, −0.18 to 11.27; *p* = 0.054). The point estimate is directionally favorable, but with only four contributing studies, moderate heterogeneity, and very low certainty, it should be treated as hypothesis-generating rather than as evidence of a proven treatment benefit.

Residual confounding is central to this interpretation. In observational practice, traction is not assigned randomly and may be selected for larger, stiffer, or more technically demanding curves. The core studies also differed in baseline curve magnitude, flexibility, fusion length, implant density, screw use, center, treatment era, and operative strategy. S001 used traction according to surgeon preference and had important baseline and instrumentation imbalances; S002 used a historical implementation design; S004 matched on several variables but retained center and metal-density differences; and S005 treated larger baseline curves in the traction group. These features can bias either toward or away from an apparent traction effect. The observed 5–6 percentage-point difference therefore should not be interpreted as a precise causal treatment effect.

The perioperative outcomes were even less stable. Number of fused levels showed no clear difference, whereas operative time and blood loss had substantial heterogeneity (*I*^2^ approximately 89%). This heterogeneity is clinically plausible because the included studies span different eras and institutional pathways. Cell-saver use, tranexamic acid dosing and timing, anesthetic technique and blood-pressure targets, transfusion thresholds, operative-team experience, implant density, osteotomy or release exposure, and the method used to measure or calculate blood loss can all materially affect these endpoints. With only three or four studies per outcome, meaningful meta-regression is not possible, so the pooled perioperative estimates should be viewed as descriptive summaries of heterogeneous practice rather than stable treatment effects.

The transfusion finding is similarly uncertain. Only two studies contributed, and although both pointed toward fewer transfusions with traction, the Hartung-Knapp 95% CI ranged from 0.03 to 6.98. The evidence therefore cannot establish that traction reduces transfusion requirements and cannot exclude no effect or harm. Future comparative studies should prospectively standardize and report cell-saver use, tranexamic acid regimens, transfusion thresholds, blood-loss definitions, operative duration, osteotomy exposure, and other co-interventions that influence transfusion.

Safety requires a separate interpretation from efficacy. A reversible IONM or MEP alert is a physiological warning signal, whereas a permanent postoperative neurological deficit is a clinical outcome; the two should not be combined or treated as interchangeable. Several studies reported substantially more alerts in traction-related contexts, including a 23.1% vs. 4.8% difference in S004 and a 53.3% traction-arm alert rate in Hadden et al. Most traction-attributed alerts recovered after reduction or removal of traction and other corrective actions, and permanent deficits were uncommon. However, the absence of permanent deficits in small, intensively monitored cohorts does not demonstrate that traction is intrinsically safe or that similar results would occur in unmonitored settings. These data support vigilant neuromonitoring and prompt traction titration when warning signals occur rather than a conclusion of neurological safety.

The high-risk morphology finding reported by Hadden et al. is a useful example. In the type-3 spinal cord morphology subgroup, all patients who received traction had IONM alerts. This should not be pooled with ordinary study-arm data because the denominator, baseline risk, and clinical setting are different. At the same time, it should not be ignored. Marked spinal cord eccentricity or reduced pericord space may reduce physiological reserve during traction-assisted correction. In these patients, longitudinal tension, local cord deformation, or changes in perfusion may be more likely to produce neuromonitoring changes. The finding supports a more individualized traction strategy that considers preoperative MRI morphology, curve rigidity, traction weight, and real-time signal response rather than applying a fixed traction protocol to all patients (18).

The present findings also differ in emphasis from earlier traction reviews because the clinical questions were different. LaMothe et al. reviewed heterogeneous pediatric scoliosis studies and concluded that intraoperative traction could be a low-morbidity adjunct, while also emphasizing the scarcity of comparative evidence (3). A meta-analysis of preoperative halo-gravity traction for severe spinal deformity found approximately 24.1% coronal correction during traction but no better final postoperative correction, illustrating that preoperative traction and intraoperative traction answer different questions (29). In neuromuscular scoliosis, Barik et al. reported a lower final Cobb angle with intraoperative traction (SMD, −0.36; 95% CI, −0.71 to 0) but nonsignificant trends for operative time and blood loss (30). In contrast, the present AIS-only analysis restricted the primary quantitative synthesis to direct intraoperative traction-vs.-no-traction comparisons and used Hartung-Knapp inference; the resulting correction estimate (MD, 5.54 percentage points; 95% CI, −0.18 to 11.27) is therefore more conservative. This evidence-layer approach avoids mixing preoperative traction, different etiologies, single-arm severe-deformity series, and neuromonitoring cohorts into a single efficacy estimate.

Several limitations remain. Only five direct comparative studies were available overall, and individual outcomes were informed by as few as two studies. Four of the five core studies were non-randomized and were judged at serious risk of bias, mainly because of residual confounding by indication and differences in treatment era, center, baseline severity, implant strategy, or other operative factors. Reporting of traction weight and duration, osteotomy and release strategy, rod material, implant density, neuromonitoring algorithms, cell-saver and tranexamic acid use, and transfusion thresholds was incomplete or inconsistent. Publication bias could not be evaluated reliably because fewer than 10 studies contributed to every pooled outcome. Finally, study-level meta-analysis cannot identify the patient subgroup or traction protocol, if any, in which a clinically meaningful net benefit exists.

These findings are insufficient to support a routine clinical recommendation for intraoperative traction in posterior AIS fusion. When traction is used as part of current practice, the available safety evidence favors a protocolized approach with reliable neuromonitoring and the ability to reduce or discontinue traction promptly in response to signal changes, but this should not be interpreted as evidence that traction improves outcomes. Prospective multicenter comparative studies with standardized traction protocols and prespecified reporting of curve severity and flexibility, traction type/weight/duration, osteotomy and release procedures, rod and implant characteristics, anesthesia and transfusion protocols, neuromonitoring thresholds, and patient-reported outcomes are needed before treatment recommendations can be made.

## 5 Conclusion

Current direct comparative evidence is insufficient to establish a statistically significant improvement in coronal correction or a reduction in transfusion requirements with intraoperative traction during posterior AIS surgery under conservative Hartung-Knapp inference. The correction point estimate favored traction, but the certainty of evidence is very low because the evidence base is sparse, clinically heterogeneous, and substantially affected by residual confounding and imprecision. Operative time, blood loss, and number of fused levels showed no clear pooled benefit. IONM/MEP alerts were more frequent in several traction-related settings and were often reversible after traction reduction or release, whereas permanent postoperative neurological deficits were uncommon; small monitored cohorts, however, cannot establish neurological safety. The evidence is therefore insufficient for routine clinical recommendation, and prospective multicenter studies using standardized traction and perioperative protocols are required.

## Data Availability

The datasets presented in this study can be found in online repositories. The names of the repository/repositories and accession number(s) can be found in the article/Supplementary Material.
