# Supplementary material for: Intraoperative traction in posterior spinal fusion for adolescent idiopathic scoliosis: an updated systematic review and meta-analysis of correction, perioperative, and safety outcomes
Source: Front Pediatr. 2026 Sep 11;14:1868371. doi: 10.3389/fped.2026.1868371 (PMC13612478; doi:10.3389/fped.2026.1868371)
Supplement: Supplementary file 1 [file Supplementaryfile1.docx]

**Supplementary Materials**

Supplementary tables and figures for the systematic review and meta-analysis of intraoperative traction in posterior spinal fusion for adolescent idiopathic scoliosis.

**Supplementary Table S1. Risk-of-bias assessment for core comparative studies.**

*Risk of bias was assessed with design-specific tools: RoB 2 for the randomized trial and ROBINS-I for the four non-randomized comparative studies. The tools are reported separately because their domains and categorical judgements are not interchangeable.*

A. RoB 2 assessment of the randomized trial.

| Study | D1 | D2 | D3 | D4 | D5 | Overall |
| --- | --- | --- | --- | --- | --- | --- |
| Hu et al., 2022 | Some concerns | Low | Low | Low | Some concerns | Some concerns |

RoB 2 domains: D1, randomization process; D2, deviations from intended interventions; D3, missing outcome data; D4, measurement of the outcome; D5, selection of the reported result. Abbreviation: RoB 2, revised Cochrane risk-of-bias tool for randomized trials. Overall judgement: some concerns because allocation-concealment and selective-reporting details were limited; follow-up was complete and radiographic assessment was independent.

B. ROBINS-I assessment of non-randomized comparative studies.

| Study | D1 | D2 | D3 | D4 | D5 | D6 | D7 | Overall |
| --- | --- | --- | --- | --- | --- | --- | --- | --- |
| Mac-Thiong et al., 2004 | Serious | Moderate | Low | Low | Moderate | Low | Moderate | Serious |
| Da Cunha et al., 2015 | Serious | Moderate | Low | Low | Low | Low | Moderate | Serious |
| Rushton et al., 2021 | Serious | Moderate | Low | Low | Low | Low | Moderate | Serious |
| Carl et al., 2023 | Serious | Moderate | Low | Low | Moderate | Low | Moderate | Serious |

ROBINS-I domains: D1, confounding; D2, selection of participants; D3, classification of interventions; D4, deviations from intended interventions; D5, missing data; D6, measurement of outcomes; D7, selection of the reported result. Abbreviations: AVT, apical vertebral translation; ROBINS-I, Risk Of Bias In Non-randomized Studies - of Interventions. Serious confounding was driven by surgeon-preference and baseline/instrumentation imbalance (S001), historical treatment era (S002), residual center/AVT/metal-density imbalance after matching (S004), and larger baseline curves with evolving surgeon experience (S005).

**Supplementary Table S2. Overall risk-of-bias summary for supplementary evidence.**

*Supplementary evidence was used for narrative, technical, or neuromonitoring safety synthesis and was not pooled in the core comparative meta-analysis.* Abbreviations: AIS, adolescent idiopathic scoliosis; IONM, intraoperative neuromonitoring; IOSFT, intraoperative skull-femoral traction; ISST, intraoperative skull-skeletal traction.

| **Study** | **Evidence role** | **Design** | **Overall judgement** | **Main limitation** | **Use in review** |
| --- | --- | --- | --- | --- | --- |
| Peiro-Garcia et al., 2019 | Supplementary comparative evidence | Retrospective cohort / quality-improvement cohort | Some concerns | Overlap with Alberta series and non-core sagittal/spinopelvic outcomes | Narrative/supplementary synthesis only |
| Bourget-Murray et al., 2019 | Supplementary resource-utilization evidence | Retrospective quality-improvement cohort | Some concerns | Cohort overlap with Da Cunha et al. and evolving technique | Not pooled with S002 |
| Lewis et al., 2011 | Neuromonitoring/safety evidence | Retrospective safety cohort | Some concerns | Safety denominator and event definitions differ from efficacy studies | Descriptive safety synthesis |
| Kato et al., 2020 | Traction-weight safety evidence | Retrospective comparative weight study | Some concerns | High- vs low-weight comparison rather than traction vs nontraction efficacy | Traction-weight safety supplement |
| Berends et al., 2024 | Neuromonitoring/safety evidence | Risk-factor study | Some concerns | Risk-factor context rather than comparative treatment effectiveness | Descriptive safety synthesis |
| Hadden et al., 2025 | Neuromonitoring/safety evidence | Risk-factor study | Some concerns | High-risk morphology subgroup not comparable with standard arms | Descriptive safety synthesis |
| Erdem et al., 2018 | Severe AIS technical evidence | Single-arm clinical series | High/serious concerns | No nontraction comparator | Narrative technical evidence |
| Jhaveri et al., 2009 | Technical/mechanistic evidence | Mixed cohort/case series | High/serious concerns | Mixed populations and noncomparative mechanism-focused evidence | Narrative mechanism evidence |
| Zhang et al., 2011 | Severe AIS technical evidence | Comparative technical cohort | Some concerns | Both treatment strategies include complex traction/staged components | Narrative technical evidence |
| Zhang et al., 2012 | Severe AIS technical evidence | Prospective comparative/quasi-randomized allocation | Some concerns | Allocation by hospitalization month; not standard RCT | Narrative technical evidence |
| Zhang/Deng et al., 2022 | Extremely severe AIS technical evidence | Single-arm case series | High/serious concerns | Preoperative plus intraoperative heavy traction and no nontraction comparator | Narrative technical evidence |
| Kulkarni et al., 2024 | Technique-comparison evidence | Comparative technical cohort | Some concerns | Both arms received intraoperative traction | Descriptive technique-comparison evidence |
| Acar et al., 2018 | Posterior temporary traction evidence | Single-arm technical study | High/serious concerns | Single-arm posterior temporary traction technique | Narrative technical evidence |

**Supplementary Table S3. GRADE summary of findings for key outcomes.**

*Certainty was assessed using GRADE domains: risk of bias, inconsistency, indirectness, imprecision, and publication bias. Following formal ROBINS-I reassessment of the non-randomized core studies, the principal comparative outcomes were rated as very-low-certainty evidence.*

| Outcome | Evidence base | Risk of bias | Inconsistency | Indirectness | Imprecision | Publication bias | Certainty |
| --- | --- | --- | --- | --- | --- | --- | --- |
| Major curve correction rate | S001, S003-S005 | Serious | Serious | Not serious | Serious | Possible | Very low |
| Operative time | S001, S003-S005 | Serious | Very serious | Not serious | Serious | Possible | Very low |
| Blood loss / EBL | S001, S003, S005 | Serious | Very serious | Not serious | Very serious | Possible | Very low |
| Blood transfusion | S002, S003 | Serious | Not serious | Not serious | Very serious | Possible | Very low |
| IONM/MEP alerts | S003, S004, S008-S011 | Serious | Serious | Serious | Serious | Possible | Very low |
| Permanent neurological deficit | Systematic review evidence | Serious | Not serious | Serious | Very serious | Possible | Very low |

Abbreviations: EBL, estimated blood loss; GRADE, Grading of Recommendations Assessment, Development and Evaluation; HK, Hartung-Knapp; IONM, intraoperative neuromonitoring; MEP, motor evoked potential. Rationale: correction was downgraded for serious confounding, heterogeneity and a Hartung-Knapp interval crossing the null; operative time and blood loss had very high heterogeneity; transfusion was based on only two studies with an HK 95% CI of 0.03-6.98; neurological safety evidence relied mainly on heterogeneous, selected, monitored cohorts with sparse permanent events.

**Supplementary Table S4. Sensitivity-analysis framework.**

*Sensitivity analyses were used to test model assumptions, study influence, and robustness of primary findings.*

| **Analysis** | **Included evidence** | **Result or handling** | **Interpretation** |
| --- | --- | --- | --- |
| Model sensitivity | Core comparative outcomes | Fixed-effect, conventional random-effects, and Hartung-Knapp random-effects estimates were compared. | Primary inference was based on Hartung-Knapp random-effects estimates. |
| Excluding S001/Mac-Thiong et al. | Correction rate, operative time, blood loss | S001 was excluded because it used head halter plus lower-extremity skin traction rather than typical skeletal traction. | Direction was checked as a prespecified sensitivity analysis. |
| Leave-one-out sensitivity | Correction rate, fused levels, operative time, blood loss | Each core study was omitted one at a time. | Continuous perioperative outcomes were less stable than correction-rate estimates. |
| SD-estimated sensitivity | Studies reporting ranges or non-SD formats | Range-derived SD estimates were treated as exploratory and were not used to define the primary pooled estimates. | Used only as a supplementary robustness check. |
| Overlapping Alberta evidence | Da Cunha et al., Peiro-Garcia et al., Bourget-Murray et al. | Da Cunha et al. was retained for the core meta-analysis; overlapping or non-core outcomes were synthesized narratively. | This avoided double-counting related cohorts. |

Abbreviations: HK, Hartung-Knapp; SD, standard deviation.

**Supplementary Table S5. Exploratory assessment of small-study effects.**

*Funnel plots were interpreted only as exploratory assessments because fewer than 10 studies were available for each pooled outcome.*

| **Outcome** | **Studies available** | **Supplementary figure panel** | **Interpretation** |
| --- | --- | --- | --- |
| Correction rate | 4 | Figure S3A | Exploratory only; fewer than 10 studies, so publication bias cannot be excluded. |
| Number of fused levels | 4 | Figure S3B | Exploratory only; fewer than 10 studies, so publication bias cannot be excluded. |
| Operative time | 4 | Figure S3C | Exploratory only; fewer than 10 studies, so publication bias cannot be excluded. |
| Blood loss | 3 | Figure S3D | Exploratory only; fewer than 10 studies, so publication bias cannot be excluded. |
| Overall complications | 3 | Figure S3E | Exploratory only; fewer than 10 studies, so publication bias cannot be excluded. |

Abbreviations: none additional; all study counts refer to the exploratory small-study-effect assessment.

**Supplementary Figure S1. Sensitivity analysis excluding S001/Mac-Thiong et al.**

*Forest plots show pooled estimates after excluding the head halter plus lower-extremity skin traction study for correction rate, operative time, and blood loss.*

**Supplementary Figure S2. Leave-one-out sensitivity analysis.**

*The plots show the pooled mean difference after omitting one study at a time for correction rate, number of fused levels, operative time, and blood loss.*

**Supplementary Figure S3. Exploratory funnel plots.**

*Contour-enhanced funnel plots are shown for correction rate, number of fused levels, operative time, blood loss, and overall complications. These plots were exploratory because fewer than 10 studies were available for each outcome.*

**Supplementary Figure S4. Risk-of-bias assessment.**

*Panel A shows RoB 2 judgements for the randomized trial. Panel B shows ROBINS-I judgements for the four non-randomized comparative studies. RoB 2 and ROBINS-I use different domain structures and category labels; the ratings are therefore displayed separately and are not combined into a single summary proportion.*
